# Bis[3-dimethyl­amino-1-(2-pyrid­yl)prop-2-en-1-one-κ^2^ *N* ^2′^,*O*]tris­(nitrato-κ^2^ *O*,*O*′)praseodymium(III)

**DOI:** 10.1107/S1600536810018817

**Published:** 2010-05-26

**Authors:** Da-Hua Hu

**Affiliations:** a Department of City Science, Jiangsu City Vocation College, Nanjing 210017, People’s Republic of China

## Abstract

In the title compound, [Pr(NO_3_)_3_(C_10_H_12_N_2_O)_2_], the Pr^III^ ion is ten-coordinated by two N and two O atoms from two bidentate 3-(dimethyl­amino)-1-(2-pyrid­yl)prop-2-en-1-one ligands and by six O atoms from three nitrate anions in a distorted bicapped square-anti­prismatic geometry. An extensive three-dimensional network of weak inter­molecular C—H⋯O hydrogen bonds consolidates the crystal packing.

## Related literature

For the crystal structures of the Co, Ni, Zn and Cd complexes with 3-(*N*,*N*-dimethyl­amino)-1-(2-pyrid­yl)prop-2-en-1-one) ligands, see: Bi (2009 ▶); Hu *et al.* (2007 ▶); Li *et al.* (2005 ▶); Wang *et al.* (2005 ▶).
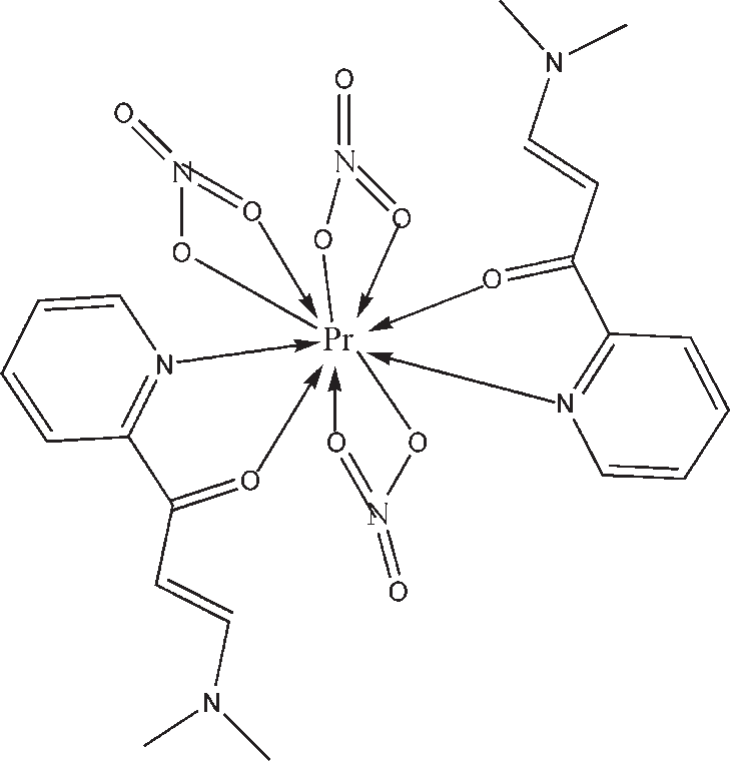


## Experimental

### 

#### Crystal data

[Pr(C_10_H_12_N_2_O)_2_(NO_3_)_3_]
*M* *_r_* = 679.37
Triclinic, 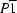

*a* = 10.2949 (10) Å
*b* = 11.2439 (11) Å
*c* = 11.7588 (12) Å
α = 92.378 (2)°
β = 108.101 (2)°
γ = 96.274 (2)°
*V* = 1281.9 (2) Å^3^
*Z* = 2
Mo *K*α radiation
μ = 1.97 mm^−1^
*T* = 291 K
0.43 × 0.26 × 0.18 mm

#### Data collection

Bruker SMART CCD area-detector diffractometer
Absorption correction: multi-scan (*SADABS*; Bruker, 2000 ▶) *T*_min_ = 0.430, *T*_max_ = 0.700
6421 measured reflections
4431 independent reflections
4090 reflections with *I* > 2σ(*I*)
*R*_int_ = 0.068

#### Refinement

*R*[*F* ^2^ > 2σ(*F* ^2^)] = 0.044
*wR*(*F* ^2^) = 0.110
*S* = 1.05
4431 reflections
356 parameters
H-atom parameters constrained
Δρ_max_ = 1.51 e Å^−3^
Δρ_min_ = −1.26 e Å^−3^

### 

Data collection: *SMART* (Bruker, 2000 ▶); cell refinement: *SAINT* (Bruker, 2000 ▶); data reduction: *SAINT*; program(s) used to solve structure: *SHELXTL* (Sheldrick, 2008 ▶); program(s) used to refine structure: *SHELXTL*; molecular graphics: *SHELXTL*; software used to prepare material for publication: *SHELXTL*.

## Supplementary Material

Crystal structure: contains datablocks I, global. DOI: 10.1107/S1600536810018817/cv2720sup1.cif

Structure factors: contains datablocks I. DOI: 10.1107/S1600536810018817/cv2720Isup2.hkl

Additional supplementary materials:  crystallographic information; 3D view; checkCIF report

## Figures and Tables

**Table 1:** Hydrogen-bond geometry (Å, °)

*D*—H⋯*A*	*D*—H	H⋯*A*	*D*⋯*A*	*D*—H⋯*A*
C2—H2⋯O4^i^	0.93	2.37	3.227 (7)	154
C3—H3⋯O7^i^	0.93	2.50	3.297 (7)	144
C4—H4⋯O10^ii^	0.93	2.58	3.174 (8)	122
C8—H8*A*⋯O11^iii^	0.93	2.52	3.377 (7)	154
C12—H12⋯O5^iv^	0.93	2.48	3.223 (8)	137
C14—H14⋯O8^v^	0.93	2.58	3.444 (7)	155
C20—H20*A*⋯O11^ii^	0.96	2.54	3.360 (9)	143
C20—H20*B*⋯O6^vi^	0.96	2.54	3.381 (7)	146
C20—H20*C*⋯O9^vi^	0.96	2.58	3.182 (8)	121

Symmetry codes: (i) 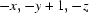; (ii) 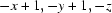; (iii) 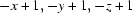; (iv) 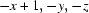; (v) 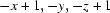; (vi) 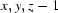.

**Fig. 1.**
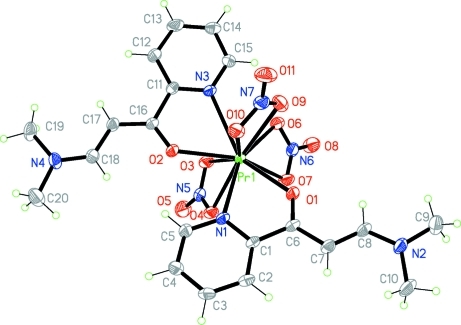
Molecular structure of the title compound, showing the atomic numbering and 30% probability displacement ellipsoids.

## supplementary crystallographic information

### Comment

Recently, the crystal structures of coordinated complexes of the ligand 3-(*N*,*N*-dimethylamino)-1-(2-pyridyl)prop-2-en-1-one) with Co, Ni, Zn and Cd were reported ((Bi, 2009; Hu *et al.*, 2007; Li *et al.*, 2005; Wang *et al.*, 2005). Here we report the crystal structure of the title complex with praseodymium(III)

The coordination geometry about Pr(III) center is shown in Fig. 1. Each Pr(III) ion is in a ten coordinate environment comprising two oxygen atoms and two nitrogen atoms from the bidentate organic ligands and six oxygen atoms from three tertiary nitrate anions that act as bidentate anion ligands. The coordination polyhedron is a distorted bicapped squareantiprism. The Pr—O distances lie in two groups, those to the oxygen atoms of organic ligands in the range 2.417 (4)-2.419 (4) (2) Å and those to nitrate O atoms in the range 2.539 (4)-2.644 (4) Å.

Weak intermolecular C—H···O hydrogen bonds (Table 1) play an important role in linking molecules into 3D supramolecular structure.

### Experimental

All solvents and chemicals were of analytical grade and were used without further purification. For the synthesis of title compoud, a solution of ligand (0.2 mmol) and Pr(NO_3_)_3_(0.1 mmol) in 50 ml of methanol was refluxed for 2 h, and then cooled to room temperature and filtered. Single crystals suitable for X-ray analysis were grown from the methanol solution by slow evaporation at room temperature in air.

### Refinement

All hydrogen atoms were geometrically positioned (C—H 0.93-0.97 Å) and refined as riding , with U_iso_(H)=1.2-1.5 U_eq_ of the parent atom.

### Crystal data

**Table:**

[Pr(C_10_H_12_N_2_O)_2_(NO_3_)_3_]	*Z* = 2
*M**_r_* = 679.37	*F*(000) = 680.0
Triclinic, *P*1	*D*_x_ = 1.760 Mg m^−^^3^
Hall symbol: -P 1	Mo *K*α radiation, λ = 0.71073 Å
*a* = 10.2949 (10) Å	Cell parameters from 4431 reflections
*b* = 11.2439 (11) Å	θ = 1.8–25.0°
*c* = 11.7588 (12) Å	µ = 1.97 mm^−^^1^
α = 92.378 (2)°	*T* = 291 K
β = 108.101 (2)°	Block, green
γ = 96.274 (2)°	0.43 × 0.26 × 0.18 mm
*V* = 1281.9 (2) Å^3^	

### Data collection

**Table:**

Bruker SMART CCD area-detector diffractometer	4431 independent reflections
Radiation source: fine-focus sealed tube	4090 reflections with *I* > 2σ(*I*)
graphite	*R*_int_ = 0.068
φ and ω scan	θ_max_ = 25.0°, θ_min_ = 1.8°
Absorption correction: multi-scan (*SADABS*; Bruker, 2000)	*h* = −11→12
*T*_min_ = 0.430, *T*_max_ = 0.700	*k* = −13→7
6421 measured reflections	*l* = −13→13

### Refinement

**Table:**

Refinement on *F*^2^	Primary atom site location: structure-invariant direct methods
Least-squares matrix: full	Secondary atom site location: difference Fourier map
*R*[*F*^2^ > 2σ(*F*^2^)] = 0.044	Hydrogen site location: inferred from neighbouring sites
*wR*(*F*^2^) = 0.110	H-atom parameters constrained
*S* = 1.05	*w* = 1/[σ^2^(*F*_o_^2^) + (0.0641*P*)^2^] where *P* = (*F*_o_^2^ + 2*F*_c_^2^)/3
4431 reflections	(Δ/σ)_max_ < 0.001
356 parameters	Δρ_max_ = 1.51 e Å^−^^3^
0 restraints	Δρ_min_ = −1.26 e Å^−^^3^

### Special details

**Table:**

Experimental. The structure was solved by direct methods (Bruker, 2000) and successive difference Fourier syntheses.
Geometry. All esds (except the esd in the dihedral angle between two l.s. planes) are estimated using the full covariance matrix. The cell esds are taken into account individually in the estimation of esds in distances, angles and torsion angles; correlations between esds in cell parameters are only used when they are defined by crystal symmetry. An approximate (isotropic) treatment of cell esds is used for estimating esds involving l.s. planes.
Refinement. Refinement of *F*^2^ against ALL reflections. The weighted *R*-factor wR and goodness of fit *S* are based on *F*^2^, conventional *R*-factors *R* are based on *F*, with *F* set to zero for negative *F*^2^. The threshold expression of *F*^2^ > σ(*F*^2^) is used only for calculating *R*-factors(gt) etc. and is not relevant to the choice of reflections for refinement. *R*-factors based on *F*^2^ are statistically about twice as large as those based on *F*, and *R*- factors based on ALL data will be even larger.

### Fractional atomic coordinates and isotropic or equivalent isotropic displacement parameters (Å^2^)

**Table:**

	*x*	*y*	*z*	*U*_iso_*/*U*_eq_	
Pr1	0.32845 (3)	0.30102 (2)	0.18779 (2)	0.03231 (12)	
N1	0.2599 (4)	0.4770 (4)	0.0429 (4)	0.0371 (10)	
N2	0.0283 (5)	0.6828 (4)	0.4065 (4)	0.0482 (12)	
N3	0.5449 (4)	0.1771 (4)	0.2475 (4)	0.0362 (9)	
N4	0.5519 (5)	0.2006 (4)	−0.2602 (4)	0.0456 (11)	
N5	0.1476 (5)	0.1201 (4)	−0.0079 (4)	0.0446 (11)	
N6	0.1779 (5)	0.1518 (4)	0.3284 (4)	0.0440 (11)	
N7	0.5768 (5)	0.4527 (4)	0.3565 (4)	0.0487 (12)	
O1	0.2248 (4)	0.4635 (3)	0.2516 (3)	0.0451 (9)	
O2	0.4446 (4)	0.2699 (3)	0.0413 (3)	0.0408 (8)	
O3	0.2390 (4)	0.0870 (3)	0.0782 (3)	0.0500 (10)	
O4	0.1341 (4)	0.2306 (3)	−0.0047 (3)	0.0474 (9)	
O5	0.0773 (5)	0.0514 (4)	−0.0920 (4)	0.0696 (13)	
O6	0.3062 (4)	0.1567 (4)	0.3466 (3)	0.0474 (9)	
O7	0.1137 (4)	0.2126 (4)	0.2498 (4)	0.0540 (10)	
O8	0.1219 (5)	0.0882 (4)	0.3868 (4)	0.0684 (12)	
O9	0.5041 (5)	0.3804 (4)	0.3969 (4)	0.0564 (11)	
O10	0.5304 (4)	0.4693 (4)	0.2469 (3)	0.0503 (10)	
O11	0.6884 (5)	0.5035 (5)	0.4183 (4)	0.0726 (14)	
C1	0.1837 (5)	0.5549 (4)	0.0720 (4)	0.0328 (10)	
C2	0.1243 (5)	0.6362 (4)	−0.0067 (5)	0.0385 (12)	
H2	0.0733	0.6910	0.0155	0.046*	
C3	0.1411 (5)	0.6355 (5)	−0.1180 (5)	0.0434 (13)	
H3	0.0988	0.6877	−0.1731	0.052*	
C4	0.2202 (6)	0.5578 (5)	−0.1471 (5)	0.0449 (13)	
H4	0.2343	0.5567	−0.2215	0.054*	
C5	0.2788 (6)	0.4812 (5)	−0.0642 (5)	0.0449 (13)	
H5	0.3347	0.4293	−0.0835	0.054*	
C6	0.1716 (5)	0.5458 (4)	0.1938 (4)	0.0343 (11)	
C7	0.1004 (5)	0.6261 (4)	0.2385 (5)	0.0406 (12)	
H7	0.0574	0.6835	0.1907	0.049*	
C8	0.0947 (5)	0.6194 (4)	0.3525 (5)	0.0406 (12)	
H8A	0.1438	0.5631	0.3972	0.049*	
C9	0.0284 (7)	0.6650 (6)	0.5285 (5)	0.0598 (17)	
H9A	0.0890	0.6072	0.5616	0.090*	
H9B	−0.0632	0.6362	0.5275	0.090*	
H9C	0.0596	0.7398	0.5769	0.090*	
C10	−0.0560 (8)	0.7710 (6)	0.3457 (7)	0.075 (2)	
H10A	−0.0149	0.8085	0.2910	0.113*	
H10B	−0.0620	0.8308	0.4039	0.113*	
H10C	−0.1467	0.7321	0.3020	0.113*	
C11	0.5974 (5)	0.1498 (4)	0.1607 (4)	0.0334 (10)	
C12	0.7071 (6)	0.0838 (5)	0.1811 (5)	0.0455 (13)	
H12	0.7430	0.0658	0.1200	0.055*	
C13	0.7617 (6)	0.0454 (5)	0.2929 (6)	0.0544 (15)	
H13	0.8365	0.0021	0.3087	0.065*	
C14	0.7062 (6)	0.0707 (5)	0.3812 (5)	0.0499 (14)	
H14	0.7402	0.0433	0.4570	0.060*	
C15	0.5993 (6)	0.1377 (5)	0.3545 (5)	0.0454 (13)	
H15	0.5625	0.1567	0.4149	0.055*	
C16	0.5313 (5)	0.1991 (4)	0.0426 (4)	0.0328 (10)	
C17	0.5716 (5)	0.1677 (4)	−0.0558 (4)	0.0373 (11)	
H17	0.6334	0.1119	−0.0495	0.045*	
C18	0.5208 (6)	0.2187 (5)	−0.1622 (4)	0.0416 (12)	
H18	0.4572	0.2720	−0.1646	0.050*	
C19	0.6499 (7)	0.1203 (5)	−0.2694 (5)	0.0561 (16)	
H19A	0.7339	0.1403	−0.2038	0.084*	
H19B	0.6686	0.1285	−0.3439	0.084*	
H19C	0.6120	0.0390	−0.2666	0.084*	
C20	0.4878 (9)	0.2591 (6)	−0.3674 (5)	0.0680 (19)	
H20A	0.4264	0.3109	−0.3512	0.102*	
H20B	0.4369	0.1995	−0.4310	0.102*	
H20C	0.5577	0.3056	−0.3910	0.102*	

### Atomic displacement parameters (Å^2^)

**Table:**

	*U*^11^	*U*^22^	*U*^33^	*U*^12^	*U*^13^	*U*^23^
Pr1	0.03546 (18)	0.03365 (18)	0.03253 (18)	0.01780 (12)	0.01260 (12)	0.00638 (11)
N1	0.039 (2)	0.036 (2)	0.042 (2)	0.0190 (19)	0.0149 (19)	0.0090 (18)
N2	0.057 (3)	0.045 (3)	0.053 (3)	0.024 (2)	0.027 (2)	0.006 (2)
N3	0.032 (2)	0.042 (2)	0.038 (2)	0.0157 (19)	0.0112 (18)	0.0101 (18)
N4	0.066 (3)	0.040 (2)	0.037 (2)	0.013 (2)	0.023 (2)	0.0030 (19)
N5	0.048 (3)	0.048 (3)	0.044 (3)	0.017 (2)	0.019 (2)	0.000 (2)
N6	0.049 (3)	0.044 (3)	0.048 (3)	0.014 (2)	0.025 (2)	0.009 (2)
N7	0.047 (3)	0.047 (3)	0.050 (3)	0.020 (2)	0.008 (2)	0.000 (2)
O1	0.061 (2)	0.041 (2)	0.043 (2)	0.0296 (19)	0.0210 (18)	0.0087 (16)
O2	0.044 (2)	0.045 (2)	0.0410 (19)	0.0234 (17)	0.0176 (16)	0.0097 (16)
O3	0.054 (2)	0.045 (2)	0.052 (2)	0.0260 (19)	0.0109 (19)	0.0069 (18)
O4	0.049 (2)	0.045 (2)	0.048 (2)	0.0237 (18)	0.0093 (18)	0.0040 (17)
O5	0.069 (3)	0.069 (3)	0.062 (3)	0.014 (2)	0.010 (2)	−0.021 (2)
O6	0.039 (2)	0.062 (2)	0.047 (2)	0.0168 (19)	0.0158 (17)	0.0141 (18)
O7	0.048 (2)	0.057 (3)	0.065 (3)	0.027 (2)	0.021 (2)	0.026 (2)
O8	0.059 (3)	0.080 (3)	0.080 (3)	0.019 (2)	0.035 (2)	0.034 (3)
O9	0.073 (3)	0.053 (2)	0.041 (2)	0.016 (2)	0.014 (2)	0.0051 (19)
O10	0.046 (2)	0.061 (3)	0.044 (2)	0.0096 (19)	0.0130 (18)	0.0104 (18)
O11	0.049 (3)	0.080 (3)	0.069 (3)	0.008 (2)	−0.009 (2)	−0.005 (2)
C1	0.023 (2)	0.032 (3)	0.044 (3)	0.007 (2)	0.011 (2)	0.004 (2)
C2	0.030 (3)	0.033 (3)	0.053 (3)	0.011 (2)	0.011 (2)	0.011 (2)
C3	0.035 (3)	0.045 (3)	0.048 (3)	0.012 (2)	0.007 (2)	0.018 (2)
C4	0.042 (3)	0.054 (3)	0.040 (3)	0.011 (3)	0.013 (2)	0.011 (2)
C5	0.052 (3)	0.049 (3)	0.042 (3)	0.025 (3)	0.020 (3)	0.011 (2)
C6	0.028 (2)	0.028 (2)	0.048 (3)	0.008 (2)	0.012 (2)	0.004 (2)
C7	0.043 (3)	0.034 (3)	0.051 (3)	0.014 (2)	0.019 (2)	0.008 (2)
C8	0.039 (3)	0.030 (3)	0.055 (3)	0.014 (2)	0.015 (2)	−0.001 (2)
C9	0.078 (5)	0.053 (4)	0.059 (4)	0.015 (3)	0.036 (3)	0.001 (3)
C10	0.094 (6)	0.073 (5)	0.082 (5)	0.059 (4)	0.044 (4)	0.020 (4)
C11	0.034 (3)	0.026 (2)	0.041 (3)	0.007 (2)	0.014 (2)	0.002 (2)
C12	0.049 (3)	0.042 (3)	0.051 (3)	0.022 (3)	0.019 (3)	0.003 (2)
C13	0.050 (4)	0.047 (3)	0.065 (4)	0.029 (3)	0.009 (3)	0.011 (3)
C14	0.047 (3)	0.053 (3)	0.049 (3)	0.020 (3)	0.008 (3)	0.016 (3)
C15	0.047 (3)	0.055 (3)	0.037 (3)	0.019 (3)	0.013 (2)	0.012 (2)
C16	0.030 (2)	0.030 (2)	0.039 (3)	0.008 (2)	0.011 (2)	0.003 (2)
C17	0.042 (3)	0.034 (3)	0.040 (3)	0.012 (2)	0.017 (2)	0.002 (2)
C18	0.046 (3)	0.040 (3)	0.041 (3)	0.013 (2)	0.016 (2)	−0.001 (2)
C19	0.080 (5)	0.045 (3)	0.059 (4)	0.018 (3)	0.042 (3)	0.000 (3)
C20	0.102 (6)	0.061 (4)	0.048 (3)	0.028 (4)	0.027 (4)	0.011 (3)

### Geometric parameters (Å, °)

**Table:**

Pr1—O2	2.417 (3)	C3—C4	1.359 (8)
Pr1—O1	2.418 (3)	C3—H3	0.9300
Pr1—O4	2.539 (4)	C4—C5	1.369 (7)
Pr1—O10	2.553 (4)	C4—H4	0.9300
Pr1—O6	2.571 (4)	C5—H5	0.9300
Pr1—O9	2.611 (4)	C6—C7	1.405 (7)
Pr1—O3	2.612 (4)	C7—C8	1.364 (8)
Pr1—O7	2.644 (4)	C7—H7	0.9300
Pr1—N1	2.675 (4)	C8—H8A	0.9300
Pr1—N3	2.681 (4)	C9—H9A	0.9600
N1—C5	1.334 (6)	C9—H9B	0.9600
N1—C1	1.338 (6)	C9—H9C	0.9600
N2—C8	1.311 (7)	C10—H10A	0.9600
N2—C10	1.452 (7)	C10—H10B	0.9600
N2—C9	1.457 (7)	C10—H10C	0.9600
N3—C15	1.326 (6)	C11—C12	1.383 (7)
N3—C11	1.335 (6)	C11—C16	1.502 (7)
N4—C18	1.302 (6)	C12—C13	1.368 (8)
N4—C19	1.450 (7)	C12—H12	0.9300
N4—C20	1.451 (7)	C13—C14	1.366 (9)
N5—O5	1.212 (6)	C13—H13	0.9300
N5—O3	1.251 (6)	C14—C15	1.365 (8)
N5—O4	1.266 (6)	C14—H14	0.9300
N6—O8	1.230 (6)	C15—H15	0.9300
N6—O7	1.237 (6)	C16—C17	1.390 (7)
N6—O6	1.265 (6)	C17—C18	1.373 (7)
N7—O11	1.216 (6)	C17—H17	0.9300
N7—O9	1.252 (7)	C18—H18	0.9300
N7—O10	1.257 (6)	C19—H19A	0.9600
O1—C6	1.245 (6)	C19—H19B	0.9600
O2—C16	1.256 (6)	C19—H19C	0.9600
C1—C2	1.382 (7)	C20—H20A	0.9600
C1—C6	1.483 (7)	C20—H20B	0.9600
C2—C3	1.373 (8)	C20—H20C	0.9600
C2—H2	0.9300		
			
O2—Pr1—O1	135.88 (12)	N7—O10—Pr1	98.1 (3)
O2—Pr1—O4	75.94 (13)	N1—C1—C2	121.3 (5)
O1—Pr1—O4	97.39 (12)	N1—C1—C6	114.2 (4)
O2—Pr1—O10	76.34 (13)	C2—C1—C6	124.5 (4)
O1—Pr1—O10	78.79 (13)	C3—C2—C1	119.4 (5)
O4—Pr1—O10	134.49 (13)	C3—C2—H2	120.3
O2—Pr1—O6	125.68 (12)	C1—C2—H2	120.3
O1—Pr1—O6	98.15 (12)	C4—C3—C2	119.4 (5)
O4—Pr1—O6	107.37 (13)	C4—C3—H3	120.3
O10—Pr1—O6	118.09 (12)	C2—C3—H3	120.3
O2—Pr1—O9	111.18 (14)	C3—C4—C5	118.4 (5)
O1—Pr1—O9	76.56 (13)	C3—C4—H4	120.8
O4—Pr1—O9	172.74 (13)	C5—C4—H4	120.8
O10—Pr1—O9	48.89 (13)	N1—C5—C4	123.4 (5)
O6—Pr1—O9	70.01 (13)	N1—C5—H5	118.3
O2—Pr1—O3	71.18 (13)	C4—C5—H5	118.3
O1—Pr1—O3	135.67 (13)	O1—C6—C7	122.9 (5)
O4—Pr1—O3	49.11 (12)	O1—C6—C1	116.8 (4)
O10—Pr1—O3	144.44 (13)	C7—C6—C1	120.3 (4)
O6—Pr1—O3	72.40 (12)	C8—C7—C6	119.5 (5)
O9—Pr1—O3	133.51 (12)	C8—C7—H7	120.2
O2—Pr1—O7	141.79 (13)	C6—C7—H7	120.2
O1—Pr1—O7	70.37 (13)	N2—C8—C7	127.7 (5)
O4—Pr1—O7	72.83 (13)	N2—C8—H8A	116.1
O10—Pr1—O7	141.87 (13)	C7—C8—H8A	116.1
O6—Pr1—O7	48.25 (12)	N2—C9—H9A	109.5
O9—Pr1—O7	101.08 (14)	N2—C9—H9B	109.5
O3—Pr1—O7	71.85 (13)	H9A—C9—H9B	109.5
O2—Pr1—N1	76.73 (12)	N2—C9—H9C	109.5
O1—Pr1—N1	61.08 (12)	H9A—C9—H9C	109.5
O4—Pr1—N1	67.04 (13)	H9B—C9—H9C	109.5
O10—Pr1—N1	71.93 (13)	N2—C10—H10A	109.5
O6—Pr1—N1	156.09 (13)	N2—C10—H10B	109.5
O9—Pr1—N1	112.44 (13)	H10A—C10—H10B	109.5
O3—Pr1—N1	113.07 (12)	N2—C10—H10C	109.5
O7—Pr1—N1	109.74 (12)	H10A—C10—H10C	109.5
O2—Pr1—N3	61.89 (12)	H10B—C10—H10C	109.5
O1—Pr1—N3	144.83 (13)	N3—C11—C12	121.4 (5)
O4—Pr1—N3	117.69 (12)	N3—C11—C16	115.0 (4)
O10—Pr1—N3	78.28 (13)	C12—C11—C16	123.5 (5)
O6—Pr1—N3	70.21 (12)	C13—C12—C11	118.9 (5)
O9—Pr1—N3	68.27 (13)	C13—C12—H12	120.6
O3—Pr1—N3	73.99 (12)	C11—C12—H12	120.6
O7—Pr1—N3	115.72 (12)	C14—C13—C12	119.9 (5)
N1—Pr1—N3	133.52 (13)	C14—C13—H13	120.1
C5—N1—C1	118.1 (4)	C12—C13—H13	120.1
C5—N1—Pr1	124.0 (3)	C15—C14—C13	117.8 (5)
C1—N1—Pr1	117.2 (3)	C15—C14—H14	121.1
C8—N2—C10	121.8 (5)	C13—C14—H14	121.1
C8—N2—C9	122.3 (5)	N3—C15—C14	123.7 (5)
C10—N2—C9	115.9 (5)	N3—C15—H15	118.1
C15—N3—C11	118.2 (4)	C14—C15—H15	118.1
C15—N3—Pr1	124.9 (3)	O2—C16—C17	124.4 (5)
C11—N3—Pr1	116.8 (3)	O2—C16—C11	116.4 (4)
C18—N4—C19	122.5 (5)	C17—C16—C11	119.2 (4)
C18—N4—C20	121.3 (5)	C18—C17—C16	120.4 (5)
C19—N4—C20	116.3 (5)	C18—C17—H17	119.8
O5—N5—O3	122.3 (5)	C16—C17—H17	119.8
O5—N5—O4	121.1 (5)	N4—C18—C17	127.2 (5)
O3—N5—O4	116.6 (4)	N4—C18—H18	116.4
O8—N6—O7	122.7 (5)	C17—C18—H18	116.4
O8—N6—O6	120.4 (5)	N4—C19—H19A	109.5
O7—N6—O6	116.9 (5)	N4—C19—H19B	109.5
O11—N7—O9	122.5 (5)	H19A—C19—H19B	109.5
O11—N7—O10	120.7 (5)	N4—C19—H19C	109.5
O9—N7—O10	116.8 (5)	H19A—C19—H19C	109.5
C6—O1—Pr1	129.3 (3)	H19B—C19—H19C	109.5
C16—O2—Pr1	128.1 (3)	N4—C20—H20A	109.5
N5—O3—Pr1	95.6 (3)	N4—C20—H20B	109.5
N5—O4—Pr1	98.7 (3)	H20A—C20—H20B	109.5
N6—O6—Pr1	98.8 (3)	N4—C20—H20C	109.5
N6—O7—Pr1	96.0 (3)	H20A—C20—H20C	109.5
N7—O9—Pr1	95.4 (3)	H20B—C20—H20C	109.5

### Hydrogen-bond geometry (Å, °)

**Table:**

*D*—H···*A*	*D*—H	H···*A*	*D*···*A*	*D*—H···*A*
C2—H2···O4^i^	0.93	2.37	3.227 (7)	154
C3—H3···O7^i^	0.93	2.50	3.297 (7)	144
C4—H4···O10^ii^	0.93	2.58	3.174 (8)	122
C8—H8A···O11^iii^	0.93	2.52	3.377 (7)	154
C12—H12···O5^iv^	0.93	2.48	3.223 (8)	137
C14—H14···O8^v^	0.93	2.58	3.444 (7)	155
C20—H20A···O11^ii^	0.96	2.54	3.360 (9)	143
C20—H20B···O6^vi^	0.96	2.54	3.381 (7)	146
C20—H20C···O9^vi^	0.96	2.58	3.182 (8)	121

Symmetry codes: (i) −*x*, −*y*+1, −*z*; (ii) −*x*+1, −*y*+1, −*z*; (iii) −*x*+1, −*y*+1, −*z*+1; (iv) −*x*+1, −*y*, −*z*; (v) −*x*+1, −*y*, −*z*+1; (vi) *x*, *y*, *z*−1.
